# Supplementary material for: Quantum Theory of Surface Lattice Resonances
Source: Nanophotonics. 2026 Feb 5;15(3):e70008. doi: 10.1002/nap2.70008 (PMC12965036; doi:10.1002/nap2.70008)
Supplement: Supplementary file 1 — Supporting Information S1 [file NAP2-15-e70008-s001.pdf]

Supplemental Material to *Quantum Theory of Surface Lattice Resonances*

M. Reitz,[1] S. v. d. Wildenberg,[1] A. Koner,[1] G. C. Schatz,[2] and J. Yuen-Zhou[1,*]

[1]*Department of Chemistry and Biochemistry, University of California San Diego, La Jolla, California 92093, USA*
[2]*Department of Chemistry, Northwestern University, Evanston, Illinois 60208, USA*

(Dated: December 16, 2025)

S1. DERIVATION OF NON-MARKOVIAN LATTICE RESPONSE

The Heisenberg equations of motion for the electric field operator and dipole amplitudes are given by

$$\dot{\hat{a}}_{\mathbf{k},\lambda} = -\mathrm{i}\omega_{\mathbf{k}}\hat{a}_{\mathbf{k},\lambda} - \mathrm{i}g^*_{\mathbf{k},\lambda}\sum_j \hat{A}_j \mathrm{e}^{-\mathrm{i}\mathbf{k}\cdot\mathbf{r}_j}, \tag{S1a}$$

$$\dot{\hat{A}}_j = -\mathrm{i}\omega_0\hat{A}_j - \mathrm{i}\sum_{\mathbf{k},\lambda} g_{\mathbf{k},\lambda}\hat{a}_{\mathbf{k},\lambda}\mathrm{e}^{\mathrm{i}\mathbf{k}\cdot\mathbf{r}_j}. \tag{S1b}$$

Formal integration of the electromagnetic field amplitudes

$$\hat{a}_{\mathbf{k},\lambda}(t) = \hat{a}_{\mathbf{k},\lambda}(0)\mathrm{e}^{-\mathrm{i}\omega_{\mathbf{k}}t} - \mathrm{i}g^*_{\mathbf{k},\lambda}\sum_{j=1}^{M}\mathrm{e}^{-\mathrm{i}\mathbf{k}\cdot\mathbf{r}_j}\int_0^t \mathrm{d}t'\,\mathrm{e}^{-\mathrm{i}\omega_{\mathbf{k}}(t-t')}\hat{A}_j(t'), \tag{S2}$$

and replacing them in the equation of motion for the dipole amplitudes yields

$$\dot{\hat{A}}_j = -\mathrm{i}\omega_0\hat{A}_j - \mathrm{i}\sum_{\mathbf{k},\lambda} g_{\mathbf{k},\lambda}\hat{a}_{\mathbf{k},\lambda}(0)\mathrm{e}^{-\mathrm{i}\omega_{\mathbf{k}}t}\mathrm{e}^{\mathrm{i}\mathbf{k}\cdot\mathbf{r}_j} - \sum_{\mathbf{k},\lambda}|g_{\mathbf{k},\lambda}|^2\sum_{j'}\mathrm{e}^{\mathrm{i}\mathbf{k}\cdot(\mathbf{r}_j-\mathbf{r}_{j'})}\int_0^t \mathrm{d}t'\mathrm{e}^{-\mathrm{i}\omega_{\mathbf{k}}(t-t')}\hat{A}_{j'}(t'). \tag{S3}$$

The second term on the right-hand side in the equation above describes the input noise acting on the MNPs which we will denote in the following simply by $\hat{A}_j^{\mathrm{in}}(t) = -\mathrm{i}\sum_{\mathbf{k},\lambda} g_{\mathbf{k},\lambda}\hat{a}_{\mathbf{k},\lambda}(0)\mathrm{e}^{-\mathrm{i}\omega_{\mathbf{k}}t}\mathrm{e}^{\mathrm{i}\mathbf{k}\cdot\mathbf{r}_j}$. We define the Fourier transformation for the dipole operators (and likewise for all other quantities) as

$$\hat{A}_j = \frac{1}{\sqrt{M}}\sum_{\mathbf{q}}\hat{A}_{\mathbf{q}}\mathrm{e}^{\mathrm{i}\mathbf{q}\cdot\mathbf{r}_j}, \qquad \hat{A}_{\mathbf{q}} = \frac{1}{\sqrt{M}}\sum_j \hat{A}_j\mathrm{e}^{-\mathrm{i}\mathbf{q}\cdot\mathbf{r}_j}, \tag{S4}$$

such that

$$\begin{aligned}\dot{\hat{A}}_{\mathbf{q}}(t) &= -\mathrm{i}\omega_0\hat{A}_{\mathbf{q}}(t) + \hat{A}^{\mathrm{in}}_{\mathbf{q}}(t) - \frac{1}{\sqrt{M}}\sum_{\mathbf{k},\lambda}|g_{\mathbf{k},\lambda}|^2\sum_{j,j'}\mathrm{e}^{-\mathrm{i}\mathbf{q}\cdot\mathbf{r}_j}\mathrm{e}^{\mathrm{i}\mathbf{k}\cdot(\mathbf{r}_j-\mathbf{r}_{j'})}\int_0^t \mathrm{d}t'\;\mathrm{e}^{-\mathrm{i}\omega_{\mathbf{k}}(t-t')}\hat{A}_{j'}(t') \\ &= -\mathrm{i}\omega_0\hat{A}_{\mathbf{q}}(t) + \hat{A}^{\mathrm{in}}_{\mathbf{q}}(t) - \sum_{\mathbf{q}'}\underbrace{\left(\frac{1}{M}\sum_{\mathbf{k},\lambda}|g_{\mathbf{k},\lambda}|^2\sum_{j,j'}\mathrm{e}^{-\mathrm{i}\mathbf{q}\cdot\mathbf{r}_j}\mathrm{e}^{\mathrm{i}\mathbf{k}\cdot(\mathbf{r}_j-\mathbf{r}_{j'})}\mathrm{e}^{\mathrm{i}\mathbf{q}'\cdot\mathbf{r}_{j'}}\right)}_{\mathcal{K}^{\mathbf{q},\mathbf{q}'}}\int_0^t \mathrm{d}t'\mathrm{e}^{-\mathrm{i}\omega_{\mathbf{k}}(t-t')}\hat{A}_{\mathbf{q}'}(t').\end{aligned} \tag{S5}$$

Let us consider only the expression in the bracket in the above equation which we denote by $\mathcal{K}^{\mathbf{q},\mathbf{q}'}$

$$\begin{aligned}\mathcal{K}^{\mathbf{q},\mathbf{q}'} &= \frac{1}{M}\sum_{\mathbf{k},\lambda}|g_{\mathbf{k},\lambda}|^2\sum_{j,j'}\mathrm{e}^{-\mathrm{i}\mathbf{q}\cdot\mathbf{r}_j}\mathrm{e}^{\mathrm{i}\mathbf{k}\cdot(\mathbf{r}_j-\mathbf{r}_{j'})}\mathrm{e}^{\mathrm{i}\mathbf{q}'\cdot\mathbf{r}_{j'}} = \\ &= \frac{1}{M}\sum_{\mathbf{k},\lambda}\frac{\omega_{\mathbf{k}}}{2\hbar\epsilon_0\mathcal{V}}|\boldsymbol{\mu}_0\cdot\boldsymbol{\varepsilon}_{\mathbf{k},\lambda}|^2\sum_{j,j'}\mathrm{e}^{-\mathrm{i}\mathbf{q}\cdot\mathbf{r}_j}\mathrm{e}^{\mathrm{i}\mathbf{k}\cdot(\mathbf{r}_j-\mathbf{r}_{j'})}\mathrm{e}^{\mathrm{i}\mathbf{q}'\cdot\mathbf{r}_{j'}}.\end{aligned} \tag{S6}$$

* joelyuen@ucsd.edu

Using the fact that $\mathbf{k} \perp \boldsymbol{\varepsilon}_{\mathbf{k},1} \perp \boldsymbol{\varepsilon}_{\mathbf{k},2}$, the sum over the polarization degree of freedom $\lambda \in \{1,2\}$ can be carried out as

$$\sum_{\lambda} |\boldsymbol{\mu}_0 \cdot \boldsymbol{\varepsilon}_{\mathbf{k},\lambda}|^2 = |\boldsymbol{\mu}_0|^2 \left(1 - (\boldsymbol{\varepsilon}_{\boldsymbol{\mu}} \cdot \boldsymbol{\varepsilon}_{\mathbf{k}})^2\right), \tag{S7}$$

where we generally denote unit vectors as $\boldsymbol{\varepsilon}_{\mathbf{v}} = \mathbf{v}/|\mathbf{v}|$ and $\boldsymbol{\varepsilon}_{\mathbf{k}}$ is now the unit vector along the direction of $\mathbf{k}$. In the limit of $\mathcal{V} \to \infty$, one can replace the discrete sum over $\mathbf{k}$ vectors with an integral and move to spherical coordinates

$$\frac{1}{\mathcal{V}} \sum_{\mathbf{k}} \to \int \frac{\mathrm{d}^3 k}{(2\pi)^3} = \frac{1}{(2\pi c)^3} \int_0^\infty \mathrm{d}\omega_{\mathbf{k}}\, \omega_{\mathbf{k}}^2 \int \mathrm{d}\Omega_{\mathbf{k}}. \tag{S8}$$

The sum can now be expressed as

$$\mathcal{K}^{\mathbf{q},\mathbf{q}'} = \frac{\mu_0^2}{2(2\pi c)^3 M \hbar \epsilon_0} \sum_{j,j'} \mathrm{e}^{-\mathrm{i}\mathbf{q}\cdot\mathbf{r}_j} \int_0^\infty \mathrm{d}\omega_{\mathbf{k}}\, \omega_{\mathbf{k}}^3 \int \mathrm{d}\Omega_{\mathbf{k}}\, [1 - (\boldsymbol{\varepsilon}_{\boldsymbol{\mu}} \cdot \boldsymbol{\varepsilon}_{\mathbf{k}})^2] \mathrm{e}^{\mathrm{i}\mathbf{k}\cdot(\mathbf{r}_j - \mathbf{r}_{j'})} \mathrm{e}^{\mathrm{i}\mathbf{q}'\cdot\mathbf{r}_j}. \tag{S9}$$

By making use of the identity $\boldsymbol{\varepsilon}_{\mathbf{k}} \mathrm{e}^{\mathrm{i}\mathbf{k}\cdot\mathbf{r}} = \nabla\, \mathrm{e}^{\mathrm{i}\mathbf{k}\cdot\mathbf{r}}/(\mathrm{i}k)$, we can substitute

$$[1 - (\boldsymbol{\varepsilon}_{\boldsymbol{\mu}} \cdot \boldsymbol{\varepsilon}_{\mathbf{k}})^2] = \left[1 + \frac{(\boldsymbol{\varepsilon}_{\boldsymbol{\mu}} \cdot \nabla)^2}{k^2}\right], \tag{S10}$$

and calculate the solid angle integral as

$$\left(1 + \frac{(\boldsymbol{\varepsilon}_{\boldsymbol{\mu}} \cdot \nabla)^2}{k^2}\right) \int_0^\pi \mathrm{d}\theta_k\, \sin\theta_k\, \mathrm{e}^{\mathrm{i}k|\mathbf{r}_j - \mathbf{r}_{j'}|\cos\theta_k} \int_0^{2\pi} \mathrm{d}\phi_k = 4\pi \left[1 + \frac{(\boldsymbol{\varepsilon}_{\boldsymbol{\mu}} \cdot \nabla)^2}{k^2}\right] \frac{\sin(k|\mathbf{r}_j - \mathbf{r}_{j'}|)}{k|\mathbf{r}_j - \mathbf{r}_{j'}|}. \tag{S11}$$

This leads us to the expression for the sum

$$\begin{aligned} \mathcal{K}^{\mathbf{q},\mathbf{q}'} &= \frac{\mu_0^2}{(2\pi)^2 c^3 \hbar \epsilon_0} \frac{1}{M} \sum_{j,j'} \mathrm{e}^{-\mathrm{i}\mathbf{q}\cdot\mathbf{r}_j} \int_0^\infty \mathrm{d}\omega_{\mathbf{k}}\, \omega_{\mathbf{k}}^3 \left[1 + \frac{(\boldsymbol{\varepsilon}_{\boldsymbol{\mu}} \cdot \nabla)^2}{k^2}\right] \frac{\sin k|\mathbf{r}_j - \mathbf{r}_{j'}|}{k|\mathbf{r}_j - \mathbf{r}_{j'}|} \mathrm{e}^{\mathrm{i}\mathbf{q}'\cdot\mathbf{r}_j} = \\ &= \int_0^\infty \mathrm{d}\omega_{\mathbf{k}}\, \frac{\mu_0^2 \omega_{\mathbf{k}}^2}{\pi c^2 \hbar \epsilon_0} \frac{1}{M} \sum_{j,j'} \mathrm{e}^{-\mathrm{i}\mathbf{q}\cdot\mathbf{r}_j} \boldsymbol{\varepsilon}_{\boldsymbol{\mu}} \cdot \mathrm{Im}[\mathbf{G}(\mathbf{r}_j, \mathbf{r}_{j'}, \omega_{\mathbf{k}})] \cdot \boldsymbol{\varepsilon}_{\boldsymbol{\mu}} \mathrm{e}^{\mathrm{i}\mathbf{q}'\cdot\mathbf{r}_{j'}}, \end{aligned} \tag{S12}$$

where we identified the imaginary part of the free space electromagnetic Green's tensor

$$\mathbf{G}(\mathbf{r}_j, \mathbf{r}_{j'}, \omega_{\mathbf{k}}) \equiv \mathbf{G}(\mathbf{r}_j - \mathbf{r}_{j'}, \omega_{\mathbf{k}}) = \left(\mathbb{1} + \frac{1}{k^2} \nabla \otimes \nabla\right) \frac{\mathrm{e}^{\mathrm{i}k|\mathbf{r}_j - \mathbf{r}_{j'}|}}{4\pi |\mathbf{r}_j - \mathbf{r}_{j'}|}, \tag{S13}$$

which can be expressed in a more practical and explicit way as

$$\mathbf{G}(\mathbf{r}, \omega_{\mathbf{k}}) = \frac{\mathrm{e}^{\mathrm{i}kr}}{4\pi k^2} \left[\left(\frac{k^2}{r} + \frac{\mathrm{i}k}{r^2} - \frac{1}{r^3}\right) \mathbb{1} + \left(-\frac{k^2}{r} - \frac{3\mathrm{i}k}{r^2} + \frac{3}{r^3}\right) \frac{\mathbf{r} \otimes \mathbf{r}}{r^2}\right]. \tag{S14}$$

We can now express Eq. (S5) as

$$\begin{aligned} \dot{\hat{A}}_{\mathbf{q}}(t) = -\mathrm{i}\omega_0 \hat{A}_{\mathbf{q}}(t) + \hat{A}_{\mathbf{q}}^{\mathrm{in}}(t) - \sum_{\mathbf{q}'} \int_0^\infty \mathrm{d}\omega_{\mathbf{k}} \int_0^t \mathrm{d}t'\, \mathrm{e}^{-\mathrm{i}\omega_{\mathbf{k}}(t-t')} \frac{\mu_0^2 \omega_{\mathbf{k}}^2}{\pi c^2 \hbar \epsilon_0} \frac{1}{M} \sum_{j,j'} \\ \times \mathrm{e}^{-\mathrm{i}\mathbf{q}\cdot\mathbf{r}_j} \boldsymbol{\varepsilon}_{\boldsymbol{\mu}} \cdot \mathrm{Im}[\mathbf{G}(\mathbf{r}_j, \mathbf{r}_{j'}, \omega_{\mathbf{k}})] \cdot \boldsymbol{\varepsilon}_{\boldsymbol{\mu}} \mathrm{e}^{\mathrm{i}\mathbf{q}'\cdot\mathbf{r}_{j'}} \hat{A}_{\mathbf{q}'}(t'). \end{aligned} \tag{S15}$$

Due to the translational invariance of the lattice, double sums can be reduced to single sums

$$\begin{aligned} \frac{1}{M} \sum_{j,j'} \mathrm{e}^{-\mathrm{i}\mathbf{q}\cdot\mathbf{r}_j} \boldsymbol{\varepsilon}_{\boldsymbol{\mu}} \cdot \mathrm{Im}[\mathbf{G}(\mathbf{r}_j, \mathbf{r}_{j'}, \omega_{\mathbf{k}})] \cdot \boldsymbol{\varepsilon}_{\boldsymbol{\mu}} \mathrm{e}^{\mathrm{i}\mathbf{q}'\cdot\mathbf{r}_{j'}} &= \frac{1}{M} \sum_{j,j'} \mathrm{e}^{-\mathrm{i}\mathbf{q}\cdot(\mathbf{r}_j - \mathbf{r}_{j'})} \mathrm{Im}\left[\mathbf{G}(\mathbf{r}_j, \mathbf{r}_{j'}, \omega_{\mathbf{k}})\right] \mathrm{e}^{\mathrm{i}(\mathbf{q}'-\mathbf{q})\cdot\mathbf{r}_{j'}} \\ &= \sum_{\Lambda} \mathrm{e}^{-\mathrm{i}\mathbf{q}\cdot\mathbf{r}_\Lambda} \boldsymbol{\varepsilon}_{\boldsymbol{\mu}} \cdot \mathrm{Im}[\mathbf{G}(\mathbf{r}_\Lambda, \omega_{\mathbf{k}})] \cdot \boldsymbol{\varepsilon}_{\boldsymbol{\mu}}\, \delta_{\mathbf{q},\mathbf{q}'}, \end{aligned} \tag{S16}$$

where the sum now only depends on all relative distances with respect to a central point in the lattice which we denote by the sum over the set of lattice displacements Λ. With this, we obtain the equation of motion

$$\dot{\hat{A}}_{\mathbf{q}}(t) = -\mathrm{i}\omega_0 \hat{A}_{\mathbf{q}}(t) + \hat{A}^{\mathrm{in}}_{\mathbf{q}}(t) - C\int_0^\infty \mathrm{d}\omega_{\mathbf{k}}\,\omega_{\mathbf{k}}^2 \int_0^t \mathrm{d}t' \mathrm{e}^{-\mathrm{i}\omega_{\mathbf{k}}(t-t')} \sum_\Lambda \mathrm{e}^{-\mathrm{i}\mathbf{q}\cdot\mathbf{r}_\Lambda} \boldsymbol{\varepsilon}_{\boldsymbol{\mu}} \cdot \mathrm{Im}[\mathbf{G}(\mathbf{r}_\Lambda, \omega_{\mathbf{k}})] \cdot \boldsymbol{\varepsilon}_{\boldsymbol{\mu}} \hat{A}_{\mathbf{q}}(t'), \tag{S17}$$

where we denoted the constant prefactors by $C = \mu_0^2/(\pi c^2 \hbar \epsilon_0)$. In Fourier space, the equation of motion for a single frequency component ω expresses as

$$\begin{aligned} -\mathrm{i}\omega \hat{A}_{\mathbf{q}}(\omega) = -\mathrm{i}\omega_0 \hat{A}_{\mathbf{q}}(\omega) + \hat{A}^{\mathrm{in}}_{\mathbf{q}}(\omega) - C\int_0^\infty \mathrm{d}\omega_{\mathbf{k}}\,\omega_{\mathbf{k}}^2 \int_0^t \mathrm{d}t' \mathrm{e}^{-\mathrm{i}\omega_{\mathbf{k}}(t-t')} \\ \times \sum_\Lambda \mathrm{e}^{-\mathrm{i}\mathbf{q}\cdot\mathbf{r}_\Lambda} \boldsymbol{\varepsilon}_{\boldsymbol{\mu}} \cdot \mathrm{Im}\left[\mathbf{G}(\mathbf{r}_\Lambda, \omega_{\mathbf{k}})\right] \cdot \boldsymbol{\varepsilon}_{\boldsymbol{\mu}} \hat{A}_{\mathbf{q}}(\omega)\, \mathrm{e}^{\mathrm{i}\omega(t-t')}. \end{aligned} \tag{S18}$$

For $t \to \infty$, making use of the Sokhotski–Plemelj theorem

$$\lim_{\epsilon\to 0^+} \int \mathrm{d}\omega_{\mathbf{k}} \int_0^\infty \mathrm{d}s\, \mathrm{e}^{-\mathrm{i}(\omega_{\mathbf{k}}-\omega_0-\mathrm{i}\epsilon)s} = \int \mathrm{d}\omega_{\mathbf{k}} \left[\pi\delta(\omega_{\mathbf{k}}-\omega_0) - \mathrm{i}\mathcal{P}\left(\frac{1}{\omega_{\mathbf{k}}-\omega_0}\right)\right], \tag{S19}$$

where $\mathcal{P}$ denotes the Cauchy principal value, and additionally making use of the (approximate) Kramers-Kronig relation for the dyadic Green's function [1–3]

$$\mathcal{P}\int_{-\infty}^\infty \mathrm{d}\omega_{\mathbf{k}}\,\omega_{\mathbf{k}}^2 \frac{\mathrm{Im}[\mathbf{G}(\mathbf{r}_\Lambda, \omega_{\mathbf{k}})]}{\omega_{\mathbf{k}} - \omega} \approx \pi\omega^2 \mathrm{Re}[\mathbf{G}(\mathbf{r}_\Lambda, \omega)], \tag{S20}$$

one finally arrives at a simple expression relating the dipole amplitudes to the input term in Fourier space

$$\begin{aligned} \hat{A}_{\mathbf{q}}(\omega) &= \left[\mathrm{i}(\omega_0 - \omega) - \mathrm{i}\frac{\mu_0^2\omega^2}{c^2\hbar\epsilon_0}\left(\sum_\Lambda \mathrm{e}^{-\mathrm{i}\mathbf{q}\cdot\mathbf{r}_\Lambda} \boldsymbol{\varepsilon}_{\boldsymbol{\mu}} \cdot \mathbf{G}(\mathbf{r}_\Lambda, \omega) \cdot \boldsymbol{\varepsilon}_{\boldsymbol{\mu}}\right)\right]^{-1} \hat{A}^{\mathrm{in}}_{\mathbf{q}}(\omega) \\ &\equiv \left[\mathrm{i}(\omega_0 - \omega) - \mathrm{i}\bar{\mathcal{S}}_{\mathbf{q}}(\omega)\right]^{-1} \hat{A}^{\mathrm{in}}_{\mathbf{q}}(\omega), \end{aligned} \tag{S21}$$

where the lattice sum

$$\bar{\mathcal{S}}_{\mathbf{q}}(\omega) = \frac{\mu_0^2\omega^2}{c^2\hbar\epsilon_0}\left(\sum_\Lambda \mathrm{e}^{-\mathrm{i}\mathbf{q}\cdot\mathbf{r}_\Lambda} \boldsymbol{\varepsilon}_{\boldsymbol{\mu}} \cdot \mathbf{G}(\mathbf{r}_\Lambda, \omega) \cdot \boldsymbol{\varepsilon}_{\boldsymbol{\mu}}\right) \tag{S22}$$

is the key quantity that one has to estimate as it describes the modification of the response due to the collective interactions among the particles in the lattice.

The imaginary part of the $\mathbf{r}_\Lambda = 0$ contribution in the sum above is well defined and gives rise to the single-particle radiative decay rate [4], while we impose the diverging real part (corresponding to the dipole self-energy of an individual particle) to vanish

$$\mathrm{i}\boldsymbol{\varepsilon}_{\boldsymbol{\mu}} \cdot \mathrm{Im}\left[\mathbf{G}(\mathbf{r}_\Lambda = 0, \omega)\right] \cdot \boldsymbol{\varepsilon}_{\boldsymbol{\mu}} = \frac{\mathrm{i}\omega}{6\pi c}, \qquad \mathrm{Re}\left[\mathbf{G}(\mathbf{r}_\Lambda = 0, \omega)\right] \equiv 0, \tag{S23}$$

such that

$$\hat{A}_{\mathbf{q}}(\omega) = \left[\mathrm{i}(\omega_0 - \omega) + \Gamma_0^{\mathrm{rad}}/2 - \mathrm{i}\mathcal{S}_{\mathbf{q}}(\omega)\right]^{-1} \hat{A}^{\mathrm{in}}_{\mathbf{q}}(\omega), \tag{S24}$$

in terms of the single-particle radiative rate $\Gamma_0^{\mathrm{rad}} = (\mu_0^2\omega_0^3)/(3\pi c^3\hbar\epsilon_0)$. Here, the reduced lattice sum $\mathcal{S}_{\mathbf{q}}(\omega)$ now excludes the zero-displacement contribution and we made a Markovian assumption for the single-particle radiative rate only by evaluating it around $\omega_0 \approx \omega$. The condition that needs to be fulfilled for SLRs is that both the real and the imaginary part of the expression in the brackets becomes (close to) zero. For numerical calculations, it is useful to

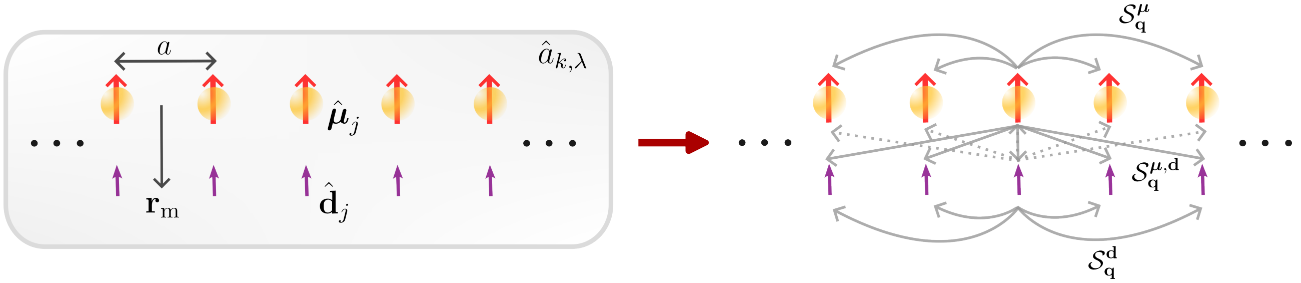

FIG. S1: **Schematics of elimination procedure.** Illustration of a nanoparticle array ($\hat{\boldsymbol{\mu}}_j$) and an array of dipolar emitters ($\hat{\mathbf{d}}_j$) which are commonly coupled to the background electromagnetic field modes $\hat{a}_{\mathbf{k},\lambda}$. Elimination of the electromagnetic field leads to effective interactions both within the arrays and between the nanoparticle and emitter array, which are encompassed in the corresponding lattice sums.

reexpress the constants appearing in front of the lattice sum in terms of the single-particle radiative decay rate as

$$\mathcal{S}_{\mathbf{q}}(\omega) = 3\pi\Gamma_0^{\mathrm{rad}} c\frac{\omega^2}{\omega_0^3}\left(\sum_{\Lambda\backslash\{0\}} \mathrm{e}^{-\mathrm{i}\mathbf{q}\cdot\mathbf{r}_\Lambda}\boldsymbol{\varepsilon}_{\boldsymbol{\mu}}\cdot\mathbf{G}(\mathbf{r}_\Lambda,\omega)\cdot\boldsymbol{\varepsilon}_{\boldsymbol{\mu}}\right) = \frac{3}{2}\Gamma_0^{\mathrm{rad}}\frac{\lambda_0^3}{\lambda(\omega)^2}\left(\sum_{\Lambda\backslash\{0\}} \mathrm{e}^{-\mathrm{i}\mathbf{q}\cdot\mathbf{r}_\Lambda}\boldsymbol{\varepsilon}_{\boldsymbol{\mu}}\cdot\mathbf{G}(\mathbf{r}_\Lambda,\omega)\cdot\boldsymbol{\varepsilon}_{\boldsymbol{\mu}}\right), \tag{S25}$$

with $\lambda(\omega) = 2\pi c/\omega$. Equivalently, we remark that one could carry out the derivation entirely in real space and apply the lattice Fourier transform only at the end.

S2. COUPLING TO A DIPOLAR ARRAY OF EMITTERS

Let us now consider the additional coupling of the nanoparticle array to an (e.g., molecular) array of dipolar emitters. We use this as an instructive case to facilitate the derivation of the optomechanical interaction in the following section. We assume the individual emitters with resonance frequency ω_d located at positions $\mathbf{r}_j^{\mathrm{m}} = \mathbf{r}_j + \mathbf{r}_{\mathrm{m}}$ described by the dipole operator $\hat{\mathbf{d}}_j = \mathbf{d}_0\left(\hat{B}_j + \hat{B}_j^\dagger\right)$ with dipole moment $\mathbf{d}_0$ and annihilation/creation operators $[\hat{B}_j, \hat{B}_{j'}^\dagger] = \delta_{j,j'}$ and typically $|\mathbf{d}_0| \ll |\boldsymbol{\mu}_0|$. We also assume that the emitter array has the same periodicity as the nanoparticle array. We start again by writing equations of motion for dipole operators as well as the electromagnetic field operators

$$\dot{\hat{a}}_{\mathbf{k},\lambda} = -\mathrm{i}\omega_{\mathbf{k}}\hat{a}_{\mathbf{k},\lambda} - \mathrm{i}g^{\boldsymbol{\mu},*}_{\mathbf{k},\lambda}\sum_j \hat{A}_j\mathrm{e}^{-\mathrm{i}\mathbf{k}\cdot\mathbf{r}_j} - \mathrm{i}g^{\mathbf{d},*}_{\mathbf{k},\lambda}\sum_j \hat{B}_j\mathrm{e}^{-\mathrm{i}\mathbf{k}\cdot\mathbf{r}_j^{\mathrm{m}}}, \tag{S26a}$$

$$\dot{\hat{A}}_j = -\mathrm{i}\omega_0\hat{A}_j - \mathrm{i}\sum_{\mathbf{k},\lambda} g^{\boldsymbol{\mu}}_{\mathbf{k},\lambda}\hat{a}_{\mathbf{k},\lambda}\mathrm{e}^{\mathrm{i}\mathbf{k}\cdot\mathbf{r}_j}, \tag{S26b}$$

$$\dot{\hat{B}}_j = -\mathrm{i}\omega_d\hat{B}_j - \mathrm{i}\sum_{\mathbf{k},\lambda} g^{\mathbf{d}}_{\mathbf{k},\lambda}\hat{a}_{\mathbf{k},\lambda}\mathrm{e}^{\mathrm{i}\mathbf{k}\cdot\mathbf{r}_j^{m}}, \tag{S26c}$$

where we denoted the couplings by $g^{\boldsymbol{\mu}}_{\mathbf{k},\lambda} = -\mathrm{i}\mathcal{E}_{\mathbf{k}}(\boldsymbol{\varepsilon}_{\mathbf{k},\lambda}\cdot\boldsymbol{\mu}_0)/\hbar$ and $g^{\mathbf{d}}_{\mathbf{k},\lambda} = -\mathrm{i}\mathcal{E}_{\mathbf{k}}(\boldsymbol{\varepsilon}_{\mathbf{k},\lambda}\cdot\mathbf{d}_0)/\hbar$. Again, we start by integrating out the electric field operators

$$\hat{a}_{\mathbf{k},\lambda}(t) = \hat{a}_{\mathbf{k},\lambda}(0)\mathrm{e}^{-\mathrm{i}\omega_{\mathbf{k}}t} - \mathrm{i}g^{\boldsymbol{\mu},*}_{\mathbf{k},\lambda}\sum_{j=1}^{M}\mathrm{e}^{-\mathrm{i}\mathbf{k}\cdot\mathbf{r}_j}\int_0^t \mathrm{d}t'\,\mathrm{e}^{-\mathrm{i}\omega_{\mathbf{k}}(t-t')}\hat{A}_j(t') - \mathrm{i}g^{\mathbf{d},*}_{\mathbf{k},\lambda}\sum_{j=1}^{M}\mathrm{e}^{-\mathrm{i}\mathbf{k}\cdot\mathbf{r}_j^{\mathrm{m}}}\int_0^t \mathrm{d}t'\,\mathrm{e}^{-\mathrm{i}\omega_{\mathbf{k}}(t-t')}\hat{B}_j(t'), \tag{S27}$$

and replacing them in the equations for the dipole operators. The procedure is now largely identical to Sec. S1 and consists in going to Fourier space both in the time and spatial domains. Finally, one ends up with an algebraic set of equations for the dipole operators

$$-\mathrm{i}\omega\hat{A}_{\mathbf{q}}(\omega) = -(\mathrm{i}\omega_0 + \Gamma_0^{\mathrm{rad}}/2)\hat{A}_{\mathbf{q}}(\omega) + \mathrm{i}\mathcal{S}^{\boldsymbol{\mu}}_{\mathbf{q}}(\omega)\hat{A}_{\mathbf{q}}(\omega) + \mathrm{i}\mathcal{S}^{\boldsymbol{\mu},\mathbf{d}}_{\mathbf{q}}(\omega)\hat{B}_{\mathbf{q}}(\omega) + \hat{A}^{\mathrm{in}}_{\mathbf{q}}(\omega), \tag{S28a}$$

$$-\mathrm{i}\omega\hat{B}_{\mathbf{q}}(\omega) = -(\mathrm{i}\omega_d + \gamma_0^{\mathrm{rad}}/2)\hat{B}_{\mathbf{q}}(\omega) + \mathrm{i}\mathcal{S}^{\mathbf{d}}_{\mathbf{q}}(\omega)\hat{B}_{\mathbf{q}}(\omega) + \mathrm{i}\mathcal{S}^{\mathbf{d},\boldsymbol{\mu}}_{\mathbf{q}}(\omega)\hat{A}_{\mathbf{q}}(\omega) + \hat{B}^{\mathrm{in}}_{\mathbf{q}}(\omega), \tag{S28b}$$

with the single-particle radiative rate of the emitters $\gamma_0^{\mathrm{rad}} = (|\mathbf{d}_0|^2\omega_d^3)/(3\pi c^3\hbar\epsilon_0)$, the lattice sums for nanoparticle and emitter lattices

$$\mathcal{S}_{\mathbf{q}}^{\boldsymbol{\mu}}(\omega) = \frac{|\boldsymbol{\mu}_0|^2\omega^2}{c^2\hbar\epsilon_0}\sum_{\Lambda\setminus\{0\}} \mathrm{e}^{-\mathrm{i}\mathbf{q}\cdot\mathbf{r}_\Lambda}\boldsymbol{\varepsilon}_{\boldsymbol{\mu}}\cdot\mathbf{G}(\mathbf{r}_\Lambda,\omega)\cdot\boldsymbol{\varepsilon}_{\boldsymbol{\mu}}, \qquad \mathcal{S}_{\mathbf{q}}^{\mathbf{d}}(\omega) = \frac{|\mathbf{d}_0|^2\omega^2}{c^2\hbar\epsilon_0}\sum_{\Lambda\setminus\{0\}} \mathrm{e}^{-\mathrm{i}\mathbf{q}\cdot\mathbf{r}_\Lambda}\boldsymbol{\varepsilon}_{\mathbf{d}}\cdot\mathbf{G}(\mathbf{r}_\Lambda,\omega)\cdot\boldsymbol{\varepsilon}_{\mathbf{d}}. \tag{S29}$$

as well as, crucially, the interaction between the nanoparticle and molecular lattices (assuming the dipole moments to be real)

$$\mathcal{S}_{\mathbf{q}}^{\boldsymbol{\mu},\mathbf{d}}(\omega) = \frac{\mu_0 d_0\omega^2}{c^2\hbar\epsilon_0}\sum_{\Lambda} \mathrm{e}^{-\mathrm{i}\mathbf{q}\cdot(\mathbf{r}_\Lambda+\mathbf{r}_{\mathrm{m}})}\boldsymbol{\varepsilon}_{\boldsymbol{\mu}}\cdot\mathbf{G}(\mathbf{r}_\Lambda+\mathbf{r}_{\mathrm{m}},\omega)\cdot\boldsymbol{\varepsilon}_{\mathbf{d}}, \tag{S30}$$

describing a sum over all interactions between nanoparticle and emitter array (see Fig. S1 for pictorial representation of lattice sums). The input noise terms $\hat{A}_{\mathbf{q}}^{\mathrm{in}}(\omega), \hat{B}_{\mathbf{q}}^{\mathrm{in}}(\omega)$ are defined analogously to Sec. S1. Assuming only the nanoparticles to be driven by a non-zero average input electric field, i.e., $\left\langle \hat{B}_{\mathrm{in}}(\omega)\right\rangle = 0$, the dipole amplitude of the nanoparticle array can be expressed as

$$\hat{A}_{\mathbf{q}}(\omega) = \frac{\mathrm{i}\boldsymbol{\mu}_0\cdot\hat{\mathbf{E}}_{\mathbf{q}}^{\mathrm{in},(-)}(\omega)}{\mathrm{i}(\omega_0-\omega)+\Gamma_0^{\mathrm{rad}}/2-\mathrm{i}\mathcal{S}_{\mathbf{q}}^{\boldsymbol{\mu}}(\omega)+\frac{\mathcal{S}_{\mathbf{q}}^{\boldsymbol{\mu},\mathbf{d}}(\omega)^2}{\mathrm{i}(\omega_d-\omega)+\gamma_0^{\mathrm{rad}}/2-\mathrm{i}\mathcal{S}_{\mathbf{q}}^{\mathbf{d}}(\omega)}}. \tag{S31}$$

S3. EXTINCITON SPECTRUM FOR DIFFERENT LATTICE CONSTANTS

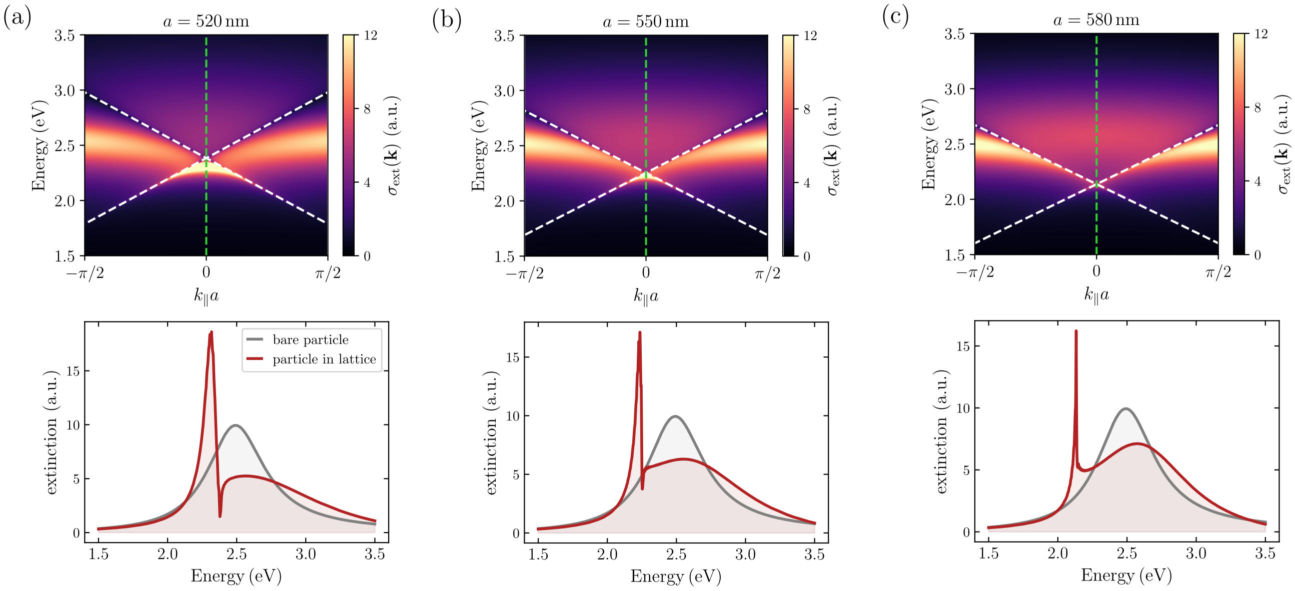

FIG. S2: **Extinction spectrum for different lattice constants.** Extinction spectra $\sigma_{\mathrm{ext}}(\mathbf{k})$ (top) as well as cross sections (illustrated by the green dashed curves) at normal incidence (bottom) for lattice constants (a) $a = 520\,\mathrm{nm}$, (b) $a = 550\,\mathrm{nm}$, and (c) $a = 580\,\mathrm{nm}$ for a resonance wavelength of $\lambda_0 = 500\,\mathrm{nm}$ and radiative linewidth $\Gamma_0 = 0.5\,\mathrm{eV}$. As the lattice constant increases, the Rayleigh anomaly shifts to longer wavelength relative to the particle resonance, resulting in a progressive narrowing of the SLR linewidth.

S4. OPTOMECHANICAL INTERACTION

In the case of molecular optomechanics, the dipoles discussed in the previous section now correspond to Raman dipoles

$$\hat{\mathcal{H}}_{\mathrm{OM}} = -\sum_j \hat{\mathbf{p}}_j^R \cdot \hat{\mathbf{E}}(\mathbf{r}_j^{\mathrm{m}}). \tag{S32}$$

The Raman dipoles are induced by the electromagnetic field acting on the molecule $\hat{\mathbf{p}}_j = \alpha_{\mathrm{m}}^j \hat{\mathbf{E}}(\mathbf{r}_j^{\mathrm{m}})$ where the proportionality is given by the molecular polarizability α_{m}^j. We assume the (scalar) polarizability to depend on a single normal-mode nuclear coordinate for each molecule $\alpha_{\mathrm{m}}^j = \alpha_{\mathrm{m}}(\hat{Q}_j)$ with $\hat{Q}_j = Q_{\mathrm{zpm}}(\hat{b}_j^\dagger + \hat{b}_j)$, where the zero-point motion is given by $Q_{\mathrm{zpm}} = \sqrt{\hbar/(2m_{\mathrm{vib}}\omega_{\mathrm{vib}})}$ with ω_{vib} and m_{vib} the vibrational frequency and reduced mass of the vibrational mode, respectively.

Due to the dependence of the Raman dipoles on the electric field, the Hamiltonian Eq. (S32) becomes quadratic in the electric field, making the treatment cumbersome. The Hamiltonian can however be linearized by the local field approximation (see, e.g., Ref. [5] where one treats the external illumination inducing the Raman dipole as a classical field at laser frequency ω_ℓ

$$\hat{\mathbf{p}}_j^R \approx \left(\mathbf{p}_j \mathrm{e}^{-\mathrm{i}\omega_\ell t} + \mathbf{p}_j^* \mathrm{e}^{\mathrm{i}\omega_\ell t}\right)(\hat{b}_j^\dagger + \hat{b}_j), \tag{S33}$$

with the classical amplitudes

$$\mathbf{p}_j = Q_{\mathrm{zpm}} \left(\frac{\partial \alpha_{\mathrm{m}}(\hat{Q}_j)}{\partial Q_j}\right)_{Q_0} \mathbf{E}(\mathbf{r}_j^m), \tag{S34}$$

and the free energy of the vibrational modes is given by $\hat{\mathcal{H}}_{\mathrm{vib}} = \sum_j \omega_{\mathrm{vib}} \hat{b}_j^\dagger \hat{b}_j$. With this, the optomechanical Hamiltonian can be written as

$$\hat{\mathcal{H}}_{\mathrm{OM}} = -\sum_j \sum_{\mathbf{k},\lambda} \mathcal{E}_{\mathbf{k}} \left(\mathbf{p}_j \mathrm{e}^{-\mathrm{i}\omega_\ell t} + \mathbf{p}_j^* \mathrm{e}^{\mathrm{i}\omega_\ell t}\right)(\hat{b}_j^\dagger + \hat{b}_j)\left(\boldsymbol{\varepsilon}_{\mathbf{k},\lambda} \hat{a}_{\mathbf{k},\lambda} \mathrm{e}^{\mathrm{i}\mathbf{k}\cdot\mathbf{r}_j^m} + \boldsymbol{\varepsilon}_{\mathbf{k},\lambda}^* \hat{a}_{\mathbf{k},\lambda}^\dagger \mathrm{e}^{-\mathrm{i}\mathbf{k}\cdot\mathbf{r}_j^m}\right). \tag{S35}$$

Under the assumption that all Raman dipoles are excited identically, i.e., $\mathbf{p}_j \equiv \mathbf{p}$ and making the RWA between Raman dipoles and electromagnetic modes which neglects simultaneous emission or absorption of two photons, one obtains

$$\hat{\mathcal{H}}_{\mathrm{OM}} = \hbar \sum_j \sum_{\mathbf{k},\lambda} \left[g_{\mathbf{k},\lambda}^{\mathrm{OM}} \hat{a}_{\mathbf{k},\lambda} \mathrm{e}^{\mathrm{i}\mathbf{k}\cdot\mathbf{r}_j^{\mathrm{m}}} \mathrm{e}^{\mathrm{i}\omega_\ell t} + g_{\mathbf{k},\lambda}^{\mathrm{OM},*} \hat{a}_{\mathbf{k},\lambda}^\dagger \mathrm{e}^{-\mathrm{i}\mathbf{k}\cdot\mathbf{r}_j^{\mathrm{m}}} \mathrm{e}^{-\mathrm{i}\omega_\ell t}\right](\hat{b}_j^\dagger + \hat{b}_j), \tag{S36}$$

with coupling $g_{\mathbf{k},\lambda}^{\mathrm{OM}} = -\mathcal{E}_{\mathbf{k}}(\boldsymbol{\varepsilon}_{\mathbf{k},\lambda} \cdot \mathbf{p}^*)/\hbar$.

Only considering the combinations in Eq. (S36) describing anti-Stokes processes (i.e., laser pump photons get up-converted in frequency aided by mechanical vibrations)

$$\hat{\mathcal{H}}_{\mathrm{OM}}^{\mathrm{AS}} \approx \hbar \sum_j \sum_{\mathbf{k},\lambda} \left[g_{\mathbf{k},\lambda}^{\mathrm{OM}} \hat{a}_{\mathbf{k},\lambda} \hat{b}_j^\dagger \mathrm{e}^{\mathrm{i}\mathbf{k}\cdot\mathbf{r}_j^{\mathrm{m}}} \mathrm{e}^{\mathrm{i}\omega_\ell t} + g_{\mathbf{k},\lambda}^{\mathrm{OM},*} \hat{a}_{\mathbf{k},\lambda}^\dagger \hat{b}_j \mathrm{e}^{-\mathrm{i}\mathbf{k}\cdot\mathbf{r}_j^{\mathrm{m}}} \mathrm{e}^{-\mathrm{i}\omega_\ell t}\right], \tag{S37}$$

leads to the equations of motion

$$\dot{\hat{a}}_{\mathbf{k},\lambda} = -\mathrm{i}\omega_{\mathbf{k}} \hat{a}_{\mathbf{k},\lambda} - \mathrm{i}\sum_j g_{\mathbf{k},\lambda}^{\boldsymbol{\mu},*} \hat{A}_j \mathrm{e}^{-\mathrm{i}\mathbf{k}\cdot\mathbf{r}_j} - \mathrm{i}\sum_j g_{\mathbf{k},\lambda}^{\mathrm{OM},*} \hat{b}_j \mathrm{e}^{-\mathrm{i}\omega_\ell t} \mathrm{e}^{-\mathrm{i}\mathbf{k}\cdot\mathbf{r}_j^{\mathrm{m}}}, \tag{S38a}$$

$$\dot{\hat{A}}_j = -\mathrm{i}\omega_0 \hat{A}_j - \mathrm{i}\sum_{\mathbf{k},\lambda} g_{\mathbf{k},\lambda}^{\boldsymbol{\mu}} \hat{a}_{\mathbf{k},\lambda} \mathrm{e}^{\mathrm{i}\mathbf{k}\cdot\mathbf{r}_j}, \tag{S38b}$$

$$\dot{\hat{b}}_j = -(\mathrm{i}\omega_{\mathrm{vib}} + \Gamma_{\mathrm{vib}}/2)\hat{b}_j - \mathrm{i}\sum_{\mathbf{k},\lambda} g_{\mathbf{k},\lambda}^{\mathrm{OM}} \hat{a}_{\mathbf{k},\lambda} \mathrm{e}^{\mathrm{i}\omega_\ell t} \mathrm{e}^{\mathrm{i}\mathbf{k}\cdot\mathbf{r}_j^{\mathrm{m}}}, \tag{S38c}$$

where we have additionally added a phenomenological vibrational damping rate Γ_{vib} in the equation of motion for $\hat{b}_j$.

To get rid of the explicit time dependence, one can go into a rotating frame by redefining $\hat{b}_j \to \hat{b}_j \mathrm{e}^{\mathrm{i}\omega_\ell t}$

$$\dot{\hat{a}}_{\mathbf{k},\lambda} = -\mathrm{i}\omega_{\mathbf{k}}\hat{a}_{\mathbf{k},\lambda} - \mathrm{i}\sum_j g^{\boldsymbol{\mu},*}_{\mathbf{k},\lambda}\hat{A}_j \mathrm{e}^{-\mathrm{i}\mathbf{k}\cdot\mathbf{r}_j} - \mathrm{i}\sum_j g^{\mathrm{OM},*}_{\mathbf{k},\lambda}\hat{b}_j \mathrm{e}^{-\mathrm{i}\mathbf{k}\cdot\mathbf{r}^{\mathrm{m}}_j}, \tag{S39a}$$

$$\dot{\hat{A}}_j = -\mathrm{i}\omega_0\hat{A}_j - \mathrm{i}\sum_{\mathbf{k},\lambda} g^{\boldsymbol{\mu}}_{\mathbf{k},\lambda}\hat{a}_{\mathbf{k},\lambda}\mathrm{e}^{\mathrm{i}\mathbf{k}\cdot\mathbf{r}_j}, \tag{S39b}$$

$$\dot{\hat{b}}_j = -\left[\mathrm{i}(\omega_{\mathrm{vib}} + \omega_\ell) + \Gamma_{\mathrm{vib}}/2\right]\hat{b}_j - \mathrm{i}\sum_{\mathbf{k},\lambda} g^{\mathrm{OM}}_{\mathbf{k},\lambda}\hat{a}_{\mathbf{k},\lambda}\mathrm{e}^{\mathrm{i}\mathbf{k}\cdot\mathbf{r}^{\mathrm{m}}_j}. \tag{S39c}$$

This is completely analogous to the case discussed in the previous section and the solution for the MNP dipole amplitude in $\mathbf{q}$ space can be expressed as

$$\hat{A}_{\mathbf{q}}(\omega) = \frac{\mathrm{i}\boldsymbol{\mu}_0\cdot\hat{\mathbf{E}}^{\mathrm{in},(-)}_{\mathbf{q}}(\omega)}{\mathrm{i}(\omega_0-\omega) + \Gamma^{\mathrm{rad}}_0/2 - \mathrm{i}\mathcal{S}^{\boldsymbol{\mu}}_{\mathbf{q}}(\omega) + \frac{\mathcal{S}^{\mathrm{OM}}_{\mathbf{q}}(\omega)^2}{\mathrm{i}(\omega_\ell+\omega_{\mathrm{vib}}-\omega)+\Gamma_{\mathrm{vib}}/2+\gamma^{\mathrm{rad}}_{\mathrm{p},+}/2-\mathrm{i}\mathcal{S}^{\mathbf{p}}_{\mathbf{q}}(\omega)}}, \tag{S40}$$

with the self-interaction of the Raman dipoles

$$\mathcal{S}^{\mathbf{p}}_{\mathbf{q}}(\omega) = \frac{|\mathbf{p}|^2\omega^2}{c^2\hbar\epsilon_0}\sum_{\Lambda\backslash\{0\}} \mathrm{e}^{-\mathrm{i}\mathbf{q}\cdot\mathbf{r}_\Lambda}\boldsymbol{\varepsilon}_{\mathbf{p}}\cdot\mathbf{G}(\mathbf{r}_\Lambda,\omega)\cdot\boldsymbol{\varepsilon}_{\mathbf{p}}, \tag{S41}$$

and the "optomechanical interaction" between the Raman dipoles and the nanoparticle dipoles

$$\mathcal{S}^{\mathrm{OM}}_{\mathbf{q}}(\omega) = \frac{\mu_0|\mathbf{p}|\omega^2}{c^2\hbar\epsilon_0}\sum_{\Lambda} \mathrm{e}^{-\mathrm{i}\mathbf{q}\cdot(\mathbf{r}_\Lambda+\mathbf{r}_{\mathrm{m}})}\boldsymbol{\varepsilon}_{\boldsymbol{\mu}}\cdot\mathbf{G}(\mathbf{r}_\Lambda+\mathbf{r}_{\mathrm{m}},\omega)\cdot\boldsymbol{\varepsilon}_{\mathbf{p}}, \tag{S42}$$

Now considering the Stokes combinations (pump photons get down-converted in frequency)

$$\hat{\mathcal{H}}^{\mathrm{S}}_{\mathrm{OM}} \approx \hbar\sum_j\sum_{\mathbf{k},\lambda}\left[g^{\mathrm{OM}}_{\mathbf{k},\lambda}\hat{a}_{\mathbf{k},\lambda}\hat{b}_j \mathrm{e}^{\mathrm{i}\mathbf{k}\cdot\mathbf{r}^{\mathrm{m}}_j}\mathrm{e}^{\mathrm{i}\omega_\ell t} + g^{\mathrm{OM},*}_{\mathbf{k},\lambda}\hat{a}^\dagger_{\mathbf{k},\lambda}\hat{b}^\dagger_j \mathrm{e}^{-\mathrm{i}\mathbf{k}\cdot\mathbf{r}^{\mathrm{m}}_j}\mathrm{e}^{-\mathrm{i}\omega_\ell t}\right], \tag{S43}$$

the equations of motion are given by

$$\dot{\hat{a}}_{\mathbf{k},\lambda} = -\mathrm{i}\omega_{\mathbf{k}}\hat{a}_{\mathbf{k},\lambda} - \mathrm{i}\sum_j g^{\boldsymbol{\mu},*}_{\mathbf{k},\lambda}\hat{A}_j \mathrm{e}^{-\mathrm{i}\mathbf{k}\cdot\mathbf{r}_j} - \mathrm{i}\sum_j g^{\mathrm{OM},*}_{\mathbf{k},\lambda}\hat{b}^\dagger_j \mathrm{e}^{-\mathrm{i}\omega_\ell t}\mathrm{e}^{-\mathrm{i}\mathbf{k}\cdot\mathbf{r}^{\mathrm{m}}_j}, \tag{S44a}$$

$$\dot{\hat{A}}_j = -\mathrm{i}\omega_0\hat{A}_j - \mathrm{i}\sum_{\mathbf{k},\lambda} g^{\boldsymbol{\mu}}_{\mathbf{k},\lambda}\hat{a}_{\mathbf{k},\lambda}\mathrm{e}^{\mathrm{i}\mathbf{k}\cdot\mathbf{r}_j}, \tag{S44b}$$

$$\dot{\hat{b}}^\dagger_j = (\mathrm{i}\omega_{\mathrm{vib}} - \Gamma_{\mathrm{vib}}/2)\hat{b}^\dagger_j + \mathrm{i}\sum_{\mathbf{k},\lambda} g^{\mathrm{OM}}_{\mathbf{k},\lambda}\hat{a}_{\mathbf{k},\lambda}\mathrm{e}^{\mathrm{i}\omega_\ell t}\mathrm{e}^{\mathrm{i}\mathbf{k}\cdot\mathbf{r}^{\mathrm{m}}_j}, \tag{S44c}$$

In a rotating frame $\hat{b}^\dagger_j \to \hat{b}^\dagger_j \mathrm{e}^{-\mathrm{i}\omega_\ell t}$, we obtain

$$\dot{\hat{a}}_{\mathbf{k},\lambda} = -\mathrm{i}\omega_{\mathbf{k}}\hat{a}_{\mathbf{k},\lambda} - \mathrm{i}\sum_j g^{\boldsymbol{\mu},*}_{\mathbf{k},\lambda}\hat{A}_j \mathrm{e}^{-\mathrm{i}\mathbf{k}\cdot\mathbf{r}_j} - \mathrm{i}\sum_j g^{\mathrm{OM},*}_{\mathbf{k},\lambda}\hat{b}^\dagger_j \mathrm{e}^{-\mathrm{i}\mathbf{k}\cdot\mathbf{r}^{\mathrm{m}}_j}, \tag{S45a}$$

$$\dot{\hat{A}}_j = -\mathrm{i}\omega_0\hat{A}_j - \mathrm{i}\sum_{\mathbf{k},\lambda} g^{\boldsymbol{\mu}}_{\mathbf{k},\lambda}\hat{a}_{\mathbf{k},\lambda}\mathrm{e}^{\mathrm{i}\mathbf{k}\cdot\mathbf{r}_j}, \tag{S45b}$$

$$\dot{\hat{b}}^\dagger_j = \left[\mathrm{i}(\omega_{\mathrm{vib}} - \omega_\ell) - \Gamma_{\mathrm{vib}}/2\right]\hat{b}^\dagger_j + \mathrm{i}\sum_{\mathbf{k},\lambda} g^{\mathrm{OM}}_{\mathbf{k},\lambda}\hat{a}_{\mathbf{k},\lambda}\mathrm{e}^{\mathrm{i}\mathbf{k}\cdot\mathbf{r}^{\mathrm{m}}_j}, \tag{S45c}$$

and the solution for the MNP dipole amplitudes is given by

$$\hat{A}_{\mathbf{q}}(\omega) = \frac{\mathrm{i}\boldsymbol{\mu}_0\cdot\hat{\mathbf{E}}^{\mathrm{in},(-)}_{\mathbf{q}}(\omega)}{\mathrm{i}(\omega_0-\omega) + \Gamma^{\mathrm{rad}}_0/2 - \mathrm{i}\mathcal{S}^{\boldsymbol{\mu}}_{\mathbf{q}}(\omega) - \frac{\mathcal{S}^{\mathrm{OM}}_{\mathbf{q}}(\omega)^2}{\mathrm{i}(\omega_\ell-\omega_{\mathrm{vib}}-\omega)+\Gamma_{\mathrm{vib}}/2-\gamma^{\mathrm{rad}}_{\mathrm{p},-}/2+\mathrm{i}\mathcal{S}^{\mathbf{p}}_{\mathbf{q}}(\omega)}}. \tag{S46}$$

The decay rates for the Raman dipoles induced by the optomechanical interaction for the anti-Stokes/Stokes sidebands are given by $\gamma_{\mathrm{p},\pm}^{\mathrm{rad}} = |\mathbf{p}|^2(\omega_\ell \pm \omega_{\mathrm{vib}})^3/(3\pi c^3\hbar\epsilon_0)$. It should be noted that the RWA for the interaction between vibrational and photonic modes has to be applied with caution, particularly in the blue-detuned regime (see discussion in main text). This is because the broad plasmonic background associated with the SLR can overlap spectrally with the anti-Stokes sideband at higher energies. In such cases, neglecting counter-rotating terms may lead to a wrong prediction of the impact of the optomechanical coupling onto the optical response. If significant overlap is present, a full non-RWA treatment is warranted to correctly describe the optical response.

We also note that fluctuations of molecular positions and phases within the unit cells can be incorporated by replacing $\mathbf{r}_\mathrm{m} \to \mathbf{r}_\mathrm{m} + \delta\mathbf{r}_{\mathrm{m},\Lambda}$, and $\mathbf{p} \to \mathbf{p}\mathrm{e}^{\mathrm{i}\Phi_\Lambda}$, which modifies the sum to

$$\mathcal{S}_{\mathbf{q}}^{\mathrm{OM}}(\omega) = \frac{\mu_0|\mathbf{p}|\omega^2}{c^2\hbar\epsilon_0}\sum_{\Lambda} \mathrm{e}^{-\mathrm{i}\mathbf{q}\cdot(\mathbf{r}_\Lambda+\mathbf{r}_\mathrm{m}+\delta\mathbf{r}_{\mathrm{m},\Lambda})}\mathrm{e}^{\mathrm{i}\Phi_\Lambda}\boldsymbol{\varepsilon}_{\boldsymbol{\mu}}\cdot\mathbf{G}(\mathbf{r}_\Lambda+\mathbf{r}_\mathrm{m}+\delta\mathbf{r}_{\mathrm{m},\Lambda},\omega)\cdot\boldsymbol{\varepsilon}_{\mathbf{p}}, \tag{S47}$$

generally leading to a renormalization of the lattice sum and therefore the optomechanical coupling. Since for most of the results presented in the main text, we focus on modes with $|\mathbf{q}| \approx 0$, whose wavelength is much larger than the lattice constant. In this long-wavelength limit, the factors $\mathrm{e}^{-\mathrm{i}\mathbf{q}\cdot\delta\mathbf{r}_{\mathrm{m},\Lambda}}$ are close to unity, such that fluctuations of the molecular positions (and similarly of the phases) are strongly suppressed in the lattice sum. As a result, these non-idealities produce only minor corrections and do not modify the qualitative behaviour discussed in the main text.

S5. NONLINEAR POLARIZABILITY OF EXCITONIC SLRS FOR STATIC POPULATIONS

Here, we derive the polarizability of the transition dipoles for an excitonic array. The interaction of the excitonic array with the electromagnetic field is described by

$$\hat{\mathcal{H}}_{\mathrm{int}} = \hbar\sum_{j,\nu<\nu'}\sum_{\mathbf{k},\lambda} g_{\mathbf{k},\lambda}^{\nu\nu'}\left(\hat{a}_{\mathbf{k},\lambda}^\dagger \mathrm{e}^{-\mathrm{i}\mathbf{k}\cdot\mathbf{r}_j}\hat{\sigma}_j^{\nu\nu'} + \mathrm{H.c.}\right), \tag{S48}$$

where $\hat{\sigma}_j^{\nu\nu'} = |\nu\rangle_j\langle\nu'|_j$ describes the lowering operator between transitions ν and ν' for a given molecule j and the coupling with the electromagnetic field is given by $g_{\mathbf{k},\lambda}^{\nu\nu'} = -\mathrm{i}\mathcal{E}_{\mathbf{k}}(\boldsymbol{\varepsilon}_{\mathbf{k},\lambda}\cdot\boldsymbol{\mu}_{\nu\nu'})/\hbar$.

One can now follow the same procedure as detailed in Sec. S1 in eliminating the photon modes to derive an effective equation for the matter part in momentum space. The dipole operator of the $\nu\nu'$-transition can be expressed as

$$-\mathrm{i}\omega\hat{\sigma}_{\mathbf{q}}^{\nu\nu'}(\omega) = -\left(\mathrm{i}\omega_{\nu\nu'} + \frac{\Gamma_{\nu\nu'}}{2}\right)\hat{\sigma}_{\mathbf{q}}^{\nu\nu'}(\omega) + \mathrm{i}\mathcal{S}_{\mathbf{q}}^{\nu\nu'}(\omega)\hat{\sigma}_{\mathbf{q}}^{\nu\nu'}(\omega)p_{\mathrm{inv}}^{\nu\nu'} - p_{\mathrm{inv}}^{\nu\nu'}\hat{\sigma}_{\mathbf{q}}^{\nu\nu',\mathrm{in}}(\omega), \tag{S49}$$

where the decay rates of the different transitions are given by $\Gamma_{\nu\nu'}^{\mathrm{rad}} = |\boldsymbol{\mu}_{\nu\nu'}|^2\omega_{\nu\nu'}^3/(3\pi c^3\hbar\epsilon_0)$. Here, the collective input noise term affecting the electronic transitions is defined as

$$\hat{\sigma}_{\mathbf{q}}^{\nu\nu',\mathrm{in}}(\omega) = -\mathrm{i}\sum_{\lambda} g_{\mathbf{q},\lambda}^{\nu\nu'}\hat{a}_{\mathbf{q},\lambda}(0)\mathrm{e}^{-\mathrm{i}\omega_{\mathbf{q}}t}. \tag{S50}$$

Importantly, here we have made the assumption of equal and constant population in all sublevels $p_{j,\mathrm{inv}}^{\nu\nu'} = \langle\hat{\sigma}_{j,\nu\nu'}^z\rangle \equiv p_{\mathrm{inv}}^{\nu\nu'}$. Furthermore, we have neglected interactions among the different subtransitions by assuming that the frequencies of the different electronic transitions are very off-resonant. The lattice sum of the $\nu\nu'$ transition dipoles is given by

$$\mathcal{S}_{\mathbf{q}}^{\nu\nu'}(\omega) = 3\pi\Gamma_{\nu\nu'}^{\mathrm{rad}}c\frac{\omega^2}{\omega_{\nu\nu'}^3}\left(\sum_{\Lambda\setminus\{0\}}\mathrm{e}^{-\mathrm{i}\mathbf{q}\cdot\mathbf{r}_\Lambda}\boldsymbol{\varepsilon}_{\boldsymbol{\mu}_{\nu\nu'}}\cdot\mathbf{G}(\mathbf{r}_\Lambda,\omega)\cdot\boldsymbol{\varepsilon}_{\boldsymbol{\mu}_{\nu\nu'}}\right). \tag{S51}$$

The solution for the dipole operator of the $\nu\nu'$ transition in Fourier space then expresses as

$$\hat{\sigma}_{\mathbf{q}}^{\nu\nu'}(\omega) = \frac{-p_{\mathrm{inv}}^{\nu\nu'}\hat{\sigma}_{\mathbf{q}}^{\nu\nu',\mathrm{in}}(\omega)}{\mathrm{i}\left(\omega_{\nu\nu'}-\omega\right) + \Gamma_{\nu\nu'}^{\mathrm{rad}}/2 + \mathrm{i}p_{\mathrm{inv}}^{\nu\nu'}\mathcal{S}_{\mathbf{q}}^{\nu\nu'}(\omega)}. \tag{S52}$$

This corresponds to the linear response result, as will be shown in the following section S5.

S6. PUMP-PROBE SPECTROSCOPY OF NONLINEAR SLR DYNAMICS

In this section, we show how to explicitly include population dynamics into our treatment. Neglecting cross-coupling between different dipole transitions, the equations of motion for the coherences express in time domain as

$$\dot{\hat{\sigma}}_j^{12}(t) = -\mathrm{i}\omega_{12}\hat{\sigma}_j^{12} - \hat{\sigma}_j^{12,\mathrm{in}}(t)\hat{p}_{\mathrm{inv},j}^{12}(t) + \hat{p}_{\mathrm{inv},j}^{12}(t)\sum_{\mathbf{k},\lambda}|g_{\mathbf{k},\lambda}^{12}|^2\sum_{j'}\int_0^t \mathrm{d}t'\mathrm{e}^{-\mathrm{i}\omega_{\mathbf{k}}(t-t')}\hat{\sigma}_{j'}^{12}(t')\mathrm{e}^{\mathrm{i}\mathbf{k}\cdot(\mathbf{r}_j-\mathbf{r}_{j'})}, \tag{S53a}$$

$$\dot{\hat{\sigma}}_j^{23}(t) = -\mathrm{i}\omega_{23}\hat{\sigma}_j^{23} - \hat{\sigma}_j^{23,\mathrm{in}}(t)\hat{p}_{\mathrm{inv},j}^{23}(t) + \hat{p}_{\mathrm{inv},j}^{23}(t)\sum_{\mathbf{k},\lambda}|g_{\mathbf{k},\lambda}^{23}|^2\sum_{j'}\int_0^t \mathrm{d}t'\mathrm{e}^{-\mathrm{i}\omega_{\mathbf{k}}(t-t')}\hat{\sigma}_{j'}^{23}(t')\mathrm{e}^{\mathrm{i}\mathbf{k}\cdot(\mathbf{r}_j-\mathbf{r}_{j'})}, \tag{S53b}$$

while the equation of motion for the population of the $|2\rangle$ state $|2\rangle_j\langle 2|_j = \hat{\sigma}_j^{12,\dagger}\hat{\sigma}_j^{12}$ is given by

$$\partial_t[\hat{\sigma}_j^{12,\dagger}\hat{\sigma}_j^{12}](t) = \hat{\sigma}_j^{12,\mathrm{in}}(t)\hat{\sigma}_j^{12,\dagger}(t) + \hat{\sigma}_j^{12,\mathrm{in}}(t)^\dagger\hat{\sigma}_j^{12}(t) - \left(\hat{\sigma}_j^{12,\dagger}(t)\sum_{\mathbf{k},\lambda}|g_{\mathbf{k},\lambda}^{12}|^2\sum_{j'}\int_0^t \mathrm{d}t'\mathrm{e}^{-\mathrm{i}\omega_{\mathbf{k}}(t-t')}\hat{\sigma}_{j'}^{12}(t')\mathrm{e}^{\mathrm{i}\mathbf{k}\cdot(\mathbf{r}_j-\mathbf{r}_{j'})} + \mathrm{H.c}\right) + \dots, \tag{S54}$$

where the ... denotes other terms which become important beyond third order for our perturbative treatment below (third-order coherences feeding back into populations) and which we therefore drop for the ease of notation.

Generally, this leads to an analytically intractable set of equations involving convolutions in the time domain and space. A simplification can be carried out by considering a perturbative expansion in terms of input amplitudes [6, 7]. To this end, we consider input pulses

$$\langle\hat{\sigma}_j^{12,\mathrm{in}}(t)\rangle = \eta_p f_p(t)\,\mathrm{e}^{-\mathrm{i}\omega_p t}\mathrm{e}^{\mathrm{i}\mathbf{k}_{\parallel,p}\cdot\mathbf{r}_j}, \qquad \langle\hat{\sigma}_j^{23,\mathrm{in}}(t)\rangle = \eta_{p'} f_{p'}(t-\tau_\Delta)\,\mathrm{e}^{-\mathrm{i}\omega_{p'} t}\mathrm{e}^{\mathrm{i}\mathbf{k}_{\parallel,p'}\cdot\mathbf{r}_j}, \tag{S55}$$

where $\eta_{p,p'}$ describes the pulse amplitudes (considered real here), $f_{p,p'}(t)$ the temporal envelope (assumed Gaussian), τ_Δ is the delay time between the pulses, and we assume incidence of pump and probe pulses at wavevectors $\mathbf{k}_{\parallel,p/p'}$. We then go to momentum space for both coherences and population operators

$$\hat{\sigma}_j^{\nu\nu'} = \frac{1}{\sqrt{M}}\sum_{\mathbf{q}}\hat{\sigma}_{\mathbf{q}}^{\nu\nu'}\mathrm{e}^{\mathrm{i}\mathbf{q}\cdot\mathbf{r}_j}, \quad [\hat{\sigma}^{\nu\nu',\dagger}\hat{\sigma}^{\nu\nu'}]_j = \frac{1}{\sqrt{M}}\sum_{\mathbf{q}}[\hat{\sigma}^{\nu\nu',\dagger}\hat{\sigma}^{\nu\nu'}]_{\mathbf{q}}\mathrm{e}^{\mathrm{i}\mathbf{q}\cdot\mathbf{r}_j}, \tag{S56}$$

and expand all operators in terms of their contribution in the input fields as

$$\langle\hat{O}_{\mathbf{q}}\rangle = \sum_{n,m=0}^{\infty}\eta_p^n\eta_{p'}^m O_{\mathbf{q}}^{(n)(m)}. \tag{S57}$$

At each order, a closed set of equations for the expansion coefficients $O_{\mathbf{q}}^{(n)(m)}$ can then be derived under the mean-field assumption $\langle\hat{\sigma}_j\hat{\sigma}_{j'}\rangle = \langle\hat{\sigma}_j\rangle\langle\hat{\sigma}_{j'}\rangle = \sigma_j\sigma_{j'}$ for $j \neq j'$ (and similarly for $\langle\hat{p}_{\mathrm{inv},j}\hat{\sigma}_{j'}\rangle$). In particular, in the following we will consider the modification of the probe response due to the second-order populations generated by the pump, corresponding to order (2)(1) [see Feynman diagrams in Fig. S3].

a. First order in pump: (1)(0). At first order in the pump field, only coherence between $|1\rangle$ and $|2\rangle$ is created and populations are static:

$$-\mathrm{i}\omega\sigma_{\mathbf{q}}^{12}(\omega)^{(1)(0)} = -\left(\mathrm{i}\omega_{12} + \frac{\Gamma_{12}^{\mathrm{rad}}}{2}\right)\sigma_{\mathbf{q}}^{12}(\omega)^{(1)(0)} - \mathrm{i}\mathcal{S}_{\mathbf{q}}^{12}(\omega)\sigma_{\mathbf{q}}^{12}(\omega)^{(1)(0)}p_{\mathrm{inv}}^{12}(0) - \delta_{\mathbf{q},\mathbf{k}_{\parallel,p}}f_p(\omega-\omega_p)p_{\mathrm{inv}}^{12}(0), \tag{S58}$$

where the populations are fixed by their initial conditions $p_{\mathrm{inv}}^{12}(0) = p_{\mathrm{inv},j}^{12}(0) = [p_{\mathrm{inv},j}^{12}]^{(0)(0)}$, and $f_p(\omega)$ denotes the Fourier transform of $f_p(t)$. For initial condition $p_{\mathrm{inv},j}^{12}(0) = -1$ (all population initially in the ground state), we obtain

$$-\mathrm{i}\omega\sigma_{\mathbf{q}}^{12}(\omega)^{(1)(0)} = -\left(\mathrm{i}\omega_{12} + \frac{\Gamma_{12}^{\mathrm{rad}}}{2}\right)\sigma_{\mathbf{q}}^{12}(\omega)^{(1)(0)} + \mathrm{i}\mathcal{S}_{\mathbf{q}}^{12}(\omega)\sigma_{\mathbf{q}}^{12}(\omega)^{(1)(0)} + \delta_{\mathbf{q},\mathbf{k}_{\parallel,p}}f_p(\omega-\omega_p). \tag{S59}$$

Therefore, at linear order only the momentum mode $\mathbf{q} = \mathbf{k}_{\parallel,p}$ (we restrict all wavevectors to the 1st Brillouin zone) is driven (and $\mathbf{q} = -\mathbf{k}_{\parallel,p}$ for the complex conjugate coherence $\sigma^{12,*}_{\mathbf{q}}$).

b. Second order in pump: (2)(0). At second order, the coherences created at first order can create populations in the $|2\rangle$ state

$$-\mathrm{i}\omega\left[\sigma^{12,\dagger}\sigma^{12}\right]_{\mathbf{q}}(\omega)^{(2)(0)} = -\Gamma^{\mathrm{rad}}_{12}\left[\sigma^{12,\dagger}\sigma^{12}\right]_{\mathbf{q}}(\omega)^{(2)(0)} - 2\mathrm{Im}\sum_{\mathbf{p}}\left[\sigma^{12,*}_{\mathbf{p}}(\omega)^{(1)(0)} * \mathcal{S}^{12}_{\mathbf{q}-\mathbf{p}}\sigma^{12}_{\mathbf{q}-\mathbf{p}}(\omega)^{(1)(0)}\right] \tag{S60}$$
$$+\left[\sigma^{12,*}_{\mathbf{q}-\mathbf{k}_{\parallel,p}}(\omega)^{(1)(0)} * f(\omega-\omega_p)\right] + \left[\sigma^{12}_{\mathbf{q}+\mathbf{k}_{\parallel,p}}(\omega)^{(1)(0)} * f(\omega-\omega_p)\right],$$

generally leading to convolutions in both momentum and frequency space. Here, an asterisk in the exponent describes complex conjugation and an asterisk between two symbols denotes convolution. Making use of the fact that the only momentum mode driven for the coherence at linear order is $\mathbf{k}_{\parallel,p}$ (and the opposite sign for the complex conjugate), we can see that only the zero-momentum mode is driven for the population at second order (see Fig. S3)

$$-\mathrm{i}\omega\left[\sigma^{12,\dagger}\sigma^{12}\right]_{\mathbf{q}=0}(\omega)^{(2)(0)} = -\Gamma^{\mathrm{rad}}_{12}\left[\sigma^{12,\dagger}\sigma^{12}\right]_{\mathbf{q}}(\omega)^{(2)(0)} - 2\mathrm{Im}\left[\sigma^{12,*}_{-\mathbf{k}_{\parallel,p}}(\omega)^{(1)(0)} * \mathcal{S}^{12}_{\mathbf{k}_{\parallel,p}}\sigma^{12}_{\mathbf{k}_{\parallel,p}}(\omega)^{(1)(0)}\right] \tag{S61}$$
$$+\left[\sigma^{12,*}_{-\mathbf{k}_{\parallel,p}}(\omega)^{(1)(0)} * f(\omega-\omega_p)\right] + \left[\sigma^{12}_{\mathbf{k}_{\parallel,p}}(\omega)^{(1)(0)} * f(\omega-\omega_p)\right].$$

c. Second order in pump, first order in probe: (2)(1). These second-order populations can now act as the generator of third-order coherences $\sigma^{23}_{\mathbf{q}}(\omega)^{(2)(1)}$ (by creating a non-zero inversion in the $2\leftrightarrow 3$ transition) via application of the probe field p':

$$-\mathrm{i}\omega\sigma^{23}_{\mathbf{q}}(\omega)^{(2)(1)} = -\left(\mathrm{i}\omega_{23} + \frac{\Gamma^{\mathrm{rad}}_{23}}{2}\right)\sigma^{12}_{\mathbf{q}}(\omega)^{(2)(1)} + \left[f_{p'}(\omega-\omega_{p'}) * \left(\sigma^{12,\dagger}\sigma^{12}\right)_{\mathbf{q}-\mathbf{k}_{\parallel,p'}}(\omega)^{(2)(0)}\right](\omega) + \mathrm{i}\mathcal{S}^{23}_{\mathbf{q}}(\omega)\sigma^{23}_{\mathbf{q}}(\omega)^{(2)(1)}. \tag{S62}$$

Therefore, the momentum mode that is driven for the coherence at third order is $\mathbf{q} = \mathbf{k}_{\parallel,p'}$, since only the population with zero quasi-momentum is contributing.

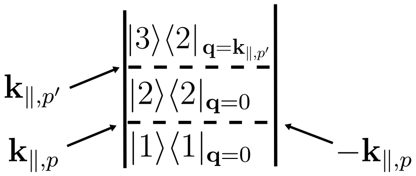

FIG. S3: **Feynman diagram of third-order process.** The pump field (p) generates zero-momentum population via two interactions with opposite phase. The probe field (p') then leads to the generation of coherence between the $|2\rangle$ and $|3\rangle$ states.

---

[1] S. Y. Buhmann, H. T. Dung, and D.-G. Welsch, "The van der Waals energy of atomic systems near absorbing and dispersing bodies," J. Opt. B: Quantum Semiclass. Opt. **6**, S127 (2004).
[2] S. Y. Buhmann and D.-G. Welsch, "Dispersion forces in macroscopic quantum electrodynamics," Prog. Quantum Electron. **31**, 51–130 (2007).
[3] D. Dzsotjan, J. Kästel, and M. Fleischhauer, "Dipole-dipole shift of quantum emitters coupled to surface plasmons of a nanowire," Phys. Rev. B **84**, 075419 (2011).
[4] L. Novotny and B. Hecht, *Principles of Nano-Optics* (Cambridge University Press, 2006).
[5] Y. Zhang, R. Esteban, R. A. Boto, M. Urbieta, X. Arrieta, C. Shan, S. Li, J. J. Baumberg, and J. Aizpurua, "Addressing molecular optomechanical effects in nanocavity-enhanced Raman scattering beyond the single plasmonic mode," Nanoscale **13**, 1938–1954 (2021).
[6] S. Mukamel, *Principles of Nonlinear Optical Spectroscopy*, Oxford series in optical and imaging sciences (Oxford University Press, New York, 1995).

[7] M. Reitz, A. Koner, and J. Yuen-Zhou, “Nonlinear semiclassical spectroscopy of ultrafast molecular polariton dynamics,” Phys. Rev. Lett. **134**, 193803 (2025).
